# A roadmap to translating the microbiome

**DOI:** 10.1186/s13073-016-0309-9

**Published:** 2016-04-28

**Authors:** Rabia Begum

**Affiliations:** Genome Medicine, BioMed Central, London, UK

In the past few decades there has been a surge in initiatives to catalogue the diversity of the human microbiome, which have been rapidly followed by research documenting the state of the microbiome in various human physiological states and geographical populations. The evidence for a causal implication of the microbiome in disease is compelling; our so-called second genome could underlie a variety of complex diseases including immunological, neurological and cardiovascular conditions, as well as cancer. It is therefore not surprising that the deep scrutiny of the normal microbiome and its intersection with disease have drawn major scientific and public interest.

In this special issue, *Genome Medicine* presents a collection of articles describing novel insights into the functional characterization of the human microbiome and the shifts in microbiome composition in disease states or recovery from disease. The issue also details the latest advances in classifying individuals according to their disease risk using computational approaches. For example, Patrick Schloss and colleagues demonstrate that a microbiota-based random forest model complements existing screening methods to detect colonic lesions; using this classification method, early stage colorectal cancer may be detected in patients in a non-invasive manner [1].

We also present the first study to develop a risk classification index for bacteremia from analysis of the fecal microbiomes of patients with non-Hodgkin lymphoma [2]. Dan Knights and colleagues present their approach to predicting the risk of chemotherapy-related blood stream infection in patients with cancer only on the basis of their pre-treatment fecal microbiome, prior to undergoing hematopoietic stem cell transplantation. The researchers conclude that patients who are likely to contract bacteremia can be identified before undergoing treatment. In a related review, Ying Taur and Eric Pamer discuss how the microbiome mediates infectious complications in patients with cancer and how to reduce infection risk in these patients [3].

These studies pave the way for translational research to exploit the therapeutic potential of modulating the microbiome. A review by Gautam Dantas and colleagues highlights the adverse effects of broad spectrum antibiotics on the dynamics between healthy and dysbiotic states; the authors expand upon the current landscape of therapeutic approaches for precision modulation of the microbiome and the advances made so far in this area [4]. In another review, Linda Zhang and Sean Davies discuss approaches to tap the therapeutic potential of key dietary metabolites that position the microbiome towards a particular disease state [5]. These insights shed light on how we can utilize the wealth of data extracted from microbiome research to maintain a healthy microbiome and prevent disease.

This special issue aims to provide a current view of the state of the field, including often forgotten components of the microbiome such as the virome [6]. Building on these collective efforts to characterize diseases and define disease risk, the field is moving towards seeking a functional and mechanistic understanding of how the microbiome produces a given phenotype. Only then can we truly understand the intimate relationship of the microbiome with health and disease, which will aid in the race to design and develop more informed diagnostic and therapeutic approaches.

We would like to express our deepest gratitude to our Guest Editor, Curtis Huttenhower, for his invaluable advice and guidance in shaping this special issue. We are also extremely grateful to the many reviewers who provided feedback and advice.
